# A comment on priors for Bayesian occupancy models

**DOI:** 10.1371/journal.pone.0192819

**Published:** 2018-02-26

**Authors:** Joseph M. Northrup, Brian D. Gerber

**Affiliations:** 1 Forest Biodiversity Research Network, Department of Forest Ecosystems and Society, Oregon State University, Corvallis, Oregon, United States of America; 2 Wildlife Research and Monitoring Section, Ontario Ministry of Natural Resources and Forestry, Peterborough, Ontario, Canada; 3 Department of Natural Resources Science, University of Rhode Island, Kingston, Rhode Island, United States of America; Southwest University, CHINA

## Abstract

Understanding patterns of species occurrence and the processes underlying these patterns is fundamental to the study of ecology. One of the more commonly used approaches to investigate species occurrence patterns is occupancy modeling, which can account for imperfect detection of a species during surveys. In recent years, there has been a proliferation of Bayesian modeling in ecology, which includes fitting Bayesian occupancy models. The Bayesian framework is appealing to ecologists for many reasons, including the ability to incorporate prior information through the specification of prior distributions on parameters. While ecologists almost exclusively intend to choose priors so that they are “uninformative” or “vague”, such priors can easily be unintentionally highly informative. Here we report on how the specification of a “vague” normally distributed (i.e., Gaussian) prior on coefficients in Bayesian occupancy models can unintentionally influence parameter estimation. Using both simulated data and empirical examples, we illustrate how this issue likely compromises inference about species-habitat relationships. While the extent to which these informative priors influence inference depends on the data set, researchers fitting Bayesian occupancy models should conduct sensitivity analyses to ensure intended inference, or employ less commonly used priors that are less informative (e.g., logistic or t prior distributions). We provide suggestions for addressing this issue in occupancy studies, and an online tool for exploring this issue under different contexts.

## Introduction

Understanding species distributions, and the environmental factors that influence occurrence is fundamental to ecology. Our knowledge of many well-studied topics in ecology, including niche partitioning, trophic interactions and metapopulation dynamics, depend on knowing which species occur in an area and why. Furthermore, occurrence patterns are critical for making conservation and management decisions; placement of reserve boundaries, or assessments of whether development will impact threatened and endangered species depend entirely on knowing whether a target species is present. Research on the patterns and drivers of species occurrence has been ongoing for many years (see [1] for a brief discussion), with major advancements over the past two decades (see [2] for a review). These advancements have stemmed from a combination of enhanced computational power, the advent of geographical information systems (GIS), and the development of a diversity of field-sampling and statistical modeling approaches that allow for detailed assessments of species habitat-relationships and the ensuing distribution patterns.

At the forefront of the methodological advancements in modeling species distributions is the explicit recognition and correction for sampling biases, such as the non-detection of a species in an area, despite it being present (i.e., false-negatives; [3]). These ‘occupancy models’ can account for the inherent imperfect detection of a species by simultaneously modeling the observation and occurrence processes. The development and refinement of these types of models has been a major focus of the ecological literature; numerous publications have developed and described occupancy models designed to address different ecological processes or sampling designs (see [4]). In addition, there now are books providing “how-to” guides [4,5] and approachable software for readily fitting occupancy models to ecological data (e.g., unmarked, MARK and PRESENCE [6–8]). These resources have allowed researchers to apply occupancy models to a range of ecological questions.

Concomitant with the increasing prevalence of occupancy models has been an increase in the use of Bayesian statistics in ecology [9–11]. The adoption of Bayesian statistics by ecologists has likely been driven by a number of factors, including the straightforward manner in which hierarchical or multi-level models can be specified and fit. Occupancy models are naturally structured hierarchically (see model below) making them straightforward to fit using Bayesian methods and there are numerous published and online resources that provide code to do so (e.g., [4,5]). The increase in the availability of these resources has made the application of Bayesian methods more approachable for practitioners and researchers.

A potential risk of the proliferation of easily accessible software and code is that researchers are perhaps fitting models without a clear understanding of the consequences of modeling choices. In Bayesian modeling, one choice that has the potential to strongly influence statistical inference is that of prior distributions [12]. Briefly, Bayesian inference focuses on summarizing posterior distributions of model parameters, which are informed jointly by the likelihood and the prior distributions. The relative influence each has on the posterior distribution depends on their information quantity. A model’s likelihood is determined entirely by the structure of the model and the data, while prior distributions represent our best knowledge about the distribution of a parameter prior to model fitting. Guidance on the choice of priors when fitting Bayesian models in ecology is limited. In our experience, researchers typically attempt to choose priors such that they are expected to have minimal or no influence on the resulting inference (i.e., “flat,” “vague,” or “uninformative” priors). Researchers commonly pick these priors so that parametric inference is primarily driven by the data, rather than the prior (e.g., [13]). However, seemingly uninformative priors often can have strong unintended consequences [14,15]. Here, we explore this issue with a specific focus on occupancy models. We show how under certain conditions, a commonly used prior can strongly influence statistical inference. We provide examples of when the use of this prior is an issue and offer guidance and alternatives when fitting Bayesian occupancy models.

### A basic Bayesian occupancy model

In the analyses and discussion below, we focus on a simple site occupancy model, formulated in a hierarchical Bayesian framework, which takes the following form,

$$y_{i}∼Binomial(n_{i},\mu_{i})$$

$$\mu_{i}=p*z_{i}$$ (1)

$$z_{i}∼Bernoulli(\psi )$$

where *y*_*i*_ indicates the number of detections at site *i*, out of a total of *n*_*i*_ sampling occasions per site, *z*_*i*_ is a latent (unobserved) parameter indicating the true occupancy state of the site (1 = occupied and 0 = unoccupied), *p* is the probability of detecting a species at the site conditional on it being occupied, and *ψ* is the probability that a site is occupied. We note that the model also can be defined in terms of species detections or non-detections in individual surveys across sites [3]. In a full Bayesian analysis, prior distributions would be specified for the unknown parameters, *p* and *ψ*. Often, uniform prior distributions between 0 and 1 are chosen (e.g., [4]).

Typically, researchers are interested in investigating hypotheses of whether environmental covariates influence species occurrence. In this case, the above model can be extended to include covariates on the occupancy process, which is most often specified as a logit regression model as,

$${logit(\psi }_{i})=\alpha +x_{i}\beta \prime$$ (2)

where *ψ*_*i*_ is the site-specific probability of occupancy, which is influenced by a matrix of covariates ***x***_***i***_ (for example, land cover type at the site), a corresponding vector of coefficients ***β*** and an intercept, *α*. The logit is a link function (i.e., log_e_($\frac{\psi }{1-\psi }$)) that takes probability values, which are restricted between 0 and 1, and projects them to values on the real number line, making estimation easier due to the lack of numerical boundaries. To recover occupancy probabilities, we use the inverse-logit of the linear combination of the intercept and covariates (i.e., logit^-1^$(\alpha +x_{i}\beta )=\frac{e^{\alpha +x_{i}\beta }}{1+e^{\alpha +x_{i}\beta }}$). We note that other link functions are available for this model, such as the probit or complementary log-log link, but in our experience, these link functions are used less in the ecological literature (though we note that use of the probit link is increasing).

### The issue of normally distributed priors in occupancy modeling

**Fig 1:**
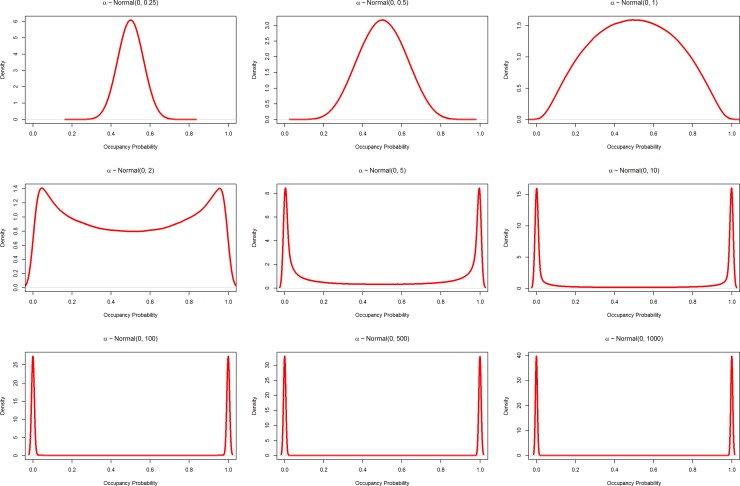
Demonstration of a Normal prior distribution transformed to the probability scale. *α* is the occupancy probability before transformation to the probability scale using the logit link; see Eq 2 in the text; panels vary by the standard deviation (*σ*) of the prior distribution. A small *σ* gives high probability density around zero, while increasing levels move this probability density towards zero and one, which eventually begins to accumulate near these values. Note that y-axes differ substantially among the panels.

The convention in Bayesian regression models is to specify normally distributed (i.e., Gaussian distribution) priors for the intercept (*α*) and coefficients (***β***), with a mean of 0 and a standard deviation (σ; e.g., α ~ Normal(0, σ) [16]; technically, in the example above a multivariate Normal prior with a vector of 0s for the mean and a covariance matrix with 0's in all the off-diagonal positions would be used for **β**). Normally distributed priors are a sensible choice for a range of reasons discussed elsewhere (e.g., [16]), but see Gelman et al. [17] for a discussion of why alternative priors might be preferred in certain cases. When conducting regression analyses, researchers typically specify these priors with a large standard deviation (*σ*) hoping to make the influence of the prior more diffuse. In simple linear regression, this choice of prior has the intended result. However, the use of such “vague” Normal priors in occupancy modeling (as well as any logistic regression model) has a potentially pernicious outcome. The issue is that the logit transformation is non-linear, such that as values become more negative or more positive, the transformed probability values approach zero and one, respectively (S1 Fig). This nonlinearity in the transformation leads to some priors that are intended to be “uninformative” becoming informative on the probability scale and strongly bimodal with large values of σ (Fig 1 and S2 Fig). It is a mathematical truism that a normally distributed prior is not invariant to this transformation—however, we believe that the consequences for modeling occupancy are not well appreciated in the ecological literature.

### Alternative prior specifications

**Fig 2:**
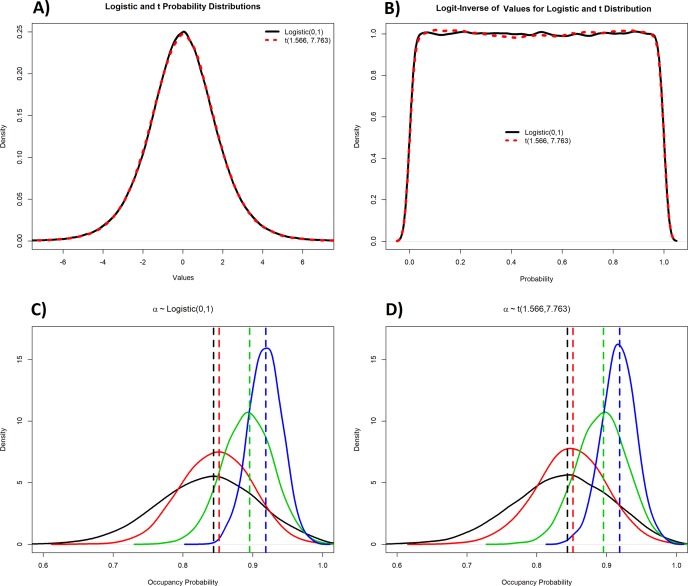
Robustness of logistic and t distribution to transformation. Panels A and B: demonstration of the Logistic and t distributions transformed to the probability scale. Panels C and D: posterior distributions (solid curves) and the corresponding maximum likelihood estimates (vertical lines) of occupancy probability from simulated data sets with varying underlying occupancy probabilities.

Numerous alternative prior specifications exist for coefficients in regression models. However, there is no consensus on prior specifications for logit-scaled parameters in logistic regression or occupancy models, and there probably should not be (see Discussion). However, the statistical and ecological literature does provide some guidance on implementing weakly informative priors. First, an exact prior distribution that is completely invariant to transformation, such as between the logit and probability scale, is the Jeffrey’s prior [18], but this prior is not uninformative and puts high probability near *ψ* = 0 and *ψ* = 1; thus a Jeffrey’s prior might not be more appropriate than a Normal distribution with small *σ*. Gelman et al. [17] suggested the use of a Cauchy distribution with center 0 and scale 2.5 as a default prior when conducting logistic regression. However, this prior still displays some bimodality at *ψ* = 0 and *ψ* = 1 and thus has the potential to affect posterior distributions. A very logical suggestion was made by Dorazio et al. [19], recommending that a weakly informative prior should assign low probability to logit-scaled values outside of -5 and 5, translating into probability values of 0.01 and 0.99 and thus approximating a Uniform(0,1) distribution for *ψ*. Their solution was to use a prior t distribution that was zero centered with scale parameter of 1.566 and degrees of freedom 7.763 (*α* ∼ *t*(*σ* = 1.566, *ν* = 7.763; Fig 2). Alternatively, a more exact specification of an implied Uniform(0,1) prior for *ψ* has been shown to be a Logistic distribution prior for *α* centered at 0 with scale parameter 1 (α~ Logistic(μ = 0, σ = 1) [20]; Fig 2). The additional benefit of the Logistic distribution is that it is easily implemented in JAGS and Winbugs. Below, we demonstrate the influence of these priors as well as the normal priors on inference from occupancy models, using both simulated and empirical datasets.

## Methods

We first demonstrate the influence of a Normal prior by simulating example occupancy datasets (using *ψ* = 0.9, *p* = 0.2, *n* =10, where *n* is the number of occasions) at a varying number of sites (50, 100, 200, and 400). For each dataset, we first fit the data in a maximum likelihood framework using the statistical program MARK [7] via the R package ‘RMark’ [21] in the programming language R [22]. Next, to illustrate the influence of the prior relative to the likelihood, we fit these models in a Bayesian framework (above model without covariates; logit(*ψ*_*i*_) = *α* and prior *α* ∼ Normal(0, *τ*) using JAGS [23] via the ‘rjags’ package ([24]; see S1 and S2 Files for example code). In JAGS, the uncertainty parameter for the Normal distribution is specified as the precision (*τ*), which is 1/*σ*^2^, where *σ* is the standard deviation of the Normal distribution. We fit the Bayesian model with normally distributed priors with *σ* values of 0.25, 0.5, 1, 2, 5, 10, 100, 500, and 1,000; algorithms were run for 10,000 Markov chain Monte Carlo (MCMC) iterations, removing the first 5,000 as a period of burn-in. Lastly, we fit the same simulated data using the t and Logistic distributions and compared posterior distributions with maximum likelihood estimates (MLE). We investigated convergence in both paradigms by fitting the models with random initial values, checking for estimate consistency. The parameters from the Bayesian analysis were also investigated for convergence by visually examining posterior distribution trace plots to ensure proper mixing and by calculating the Gelman-Rubin diagnostic [25] to ensure values were close to 1, which they always were. We compare the likelihood results, which are not influenced by the assumed prior distribution, with the Bayesian results by plotting posterior distributions of *ψ* for each dataset and prior, along with MLE. Assuming convergence and a sufficiently large number of samples, the discrepancy between the posterior mode (i.e., most probable value) and the MLE is a consequence of the assumed prior. We note that our focus is different from many simulation studies, where the aim is to evaluate the discrepancies between estimated and true parameter values. Here, we are strictly interested in unintended consequences of prior specifications and its influence on parametric inference.

We further illustrate this issue by fitting occupancy models to empirical avian point count data collected by McGarigal and McComb [26]. The authors visited over 1,000 sites in the Oregon Coast Range, USA, four times between 1990 and 1992. We fit the basic occupancy model with covariates to detections for three species: gray jay (*Perisoreus Canadensis*), Steller’s jay (*Cyanocitta stelleri*) and song sparrow (*Melospiza melodia*) with two covariates: the first representing the distance to forest edges, and the other representing the proportion of the area within 1,000 m comprised of mature forest (derived from a gradient nearest neighbor method; [27]). We specified a Uniform prior on detection probability (p ~ Uniform(0,1)), and a normally distributed prior on the intercept and coefficients of occupancy probability with a mean of 0 and *σ*^2^ ranging between 1 and 1,000 (1, 10, 100, and 1,000). We standardized both covariates (subtracting the mean and dividing by the standard deviation), and fit the Bayesian occupancy model with 10,000 MCMC iterations, dropping the first 5,000 as burn-in. Diagnosis of convergence followed the same procedure outlined above for the simulated datasets. We also fit each model in MARK, using the ‘RMark’ package. For comparison, we fit the same data using the t and Logistic prior distributions.

To assess the relative prevalence of the use of informative Normal priors in the ecological literature we performed a review of the use of priors in Bayesian occupancy modeling. We searched for articles published since 2010 using the term “Bayesian occupancy model” on Web of Science (http://apps.webofknowledge.com). We filtered results to include only those articles published in the field of ecology. We further eliminated any articles that focused on the development and refinement of methods for fitting occupancy models. We reviewed a random sample of 55 of the remaining 108 articles, attempting to identify the priors specified.

## Results

**Fig 3:**
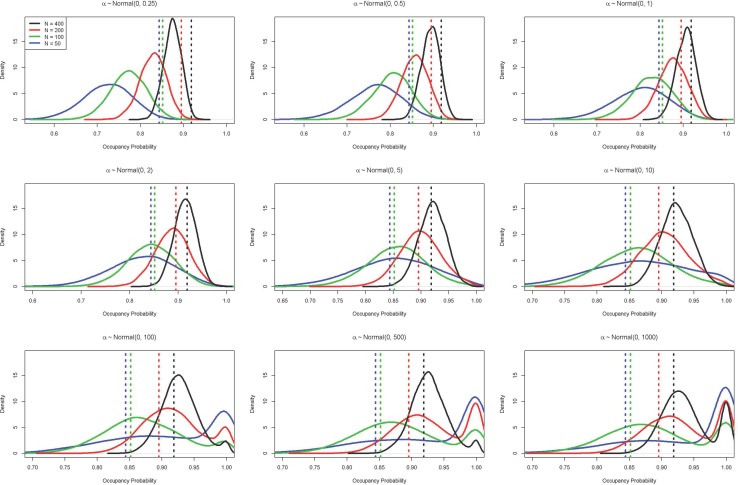
Effects of prior variance on posterior occupancy probability. Posterior distributions (solid curves) and the corresponding maximum likelihood estimates (vertical lines) of occupancy probability from simulated data sets of varying number of sites (N = 50, 100, 200, and 400); data were simulated with a true occupancy of 0.9, a per occasion detection probability of 0.2 and 10 sampling occasions. If prior distributions were truly uninformative, the posterior mode would correspond to the maximum likelihood estimate.

For the simulated datasets with normally distributed priors, when the prior standard deviation was small (i.e., σ < 2) the posterior mode was always smaller than the MLE (Fig 3), as these priors drew the posterior towards a probability of 0.5. With a standard deviation of 2, the posterior mode was approximately the MLE. At intermediate values of the standard deviation (between 5 and 10), the posterior mode was close to the MLE, but the proximity was influenced by the number of surveyed sites (Fig 3). As *σ* became large (>100), the posterior became bimodal, with one mode close to the MLE and the other close to 1 (Fig 2). Importantly, having a large number of sampled sites only mitigated the influence of the prior in a relatively narrow band of values. Generally, the nature of the influence of the prior on the posterior and subsequent ecological inference depends on a combination of effects including: 1) the true underlying detection and occupancy probabilities; 2) the number of sampled sites; 3) the number of surveys per site; and, 4) the linear combination of coefficients and covariates. Importantly, the linear combination (i.e., *α* + ***x***_***i***_***β***′) is the quantity that is transformed, and thus in some cases very large magnitude values for coefficients, when combined with certain values of covariates, could lead to scenarios where the transformation is not impactful (e.g., when there is a strong effect of a covariate that ranges over a very small set of values). However, in other cases, the use of Normal prior distributions with a large *σ* can seriously affect parametric estimates. Occupancy models using the Logistic and t distribution priors estimated posterior modes corresponding to the MLE under all sample sizes (Fig 2).

In our empirical analysis, song sparrows were detected at 271 of 1,046 sites, Steller’s jays were detected at 297 sites, and gray jays were detected at 109 sites. We found clear effects of the prior for the gray jay and Steller’s jay data, but not the song sparrow. For both jay species, the median and upper credible bound increased with the prior variance (*σ*^2^), while for the song sparrow, there were no apparent effects of the prior on the posterior (S1 Table). For the jays, estimates approaching the MLE could only be obtained by fine-tuning the prior iteratively (results not shown here). Thus, for these datasets, there are very few specifications of the Normal prior distribution that will not impact the posterior distribution and thus our inference on occupancy. Further, many more MCMC iterations were needed to achieve convergence for the gray jay model fit with values of σ^2^ greater than 10, likely due to bimodality similar to that seen in the analysis of simulated datasets (S3 Fig). In addition to causing issues with convergence, the gray jay results also highlight how these priors can impact inference on the habitat factors influencing occupancy. Whether or not credible intervals include 0 is often taken as evidence for the existence of an effect of a covariate on occupancy (though we note that Bayesian analyses provide full posterior distributions for the probability of a parameter conditional on the data, and thus much more information about parameters than is given when solely reporting the 95% credible intervals). For Steller’s jays, the 95% credible intervals (and 95% confidence intervals for the MARK analysis) for the effect of mature forest and edges all overlapped 0, indicating weak evidence for an effect. However, for gray jays the credible intervals (and confidence intervals for the MARK analysis) did not overlap 0 except in the Bayesian analysis when σ^2^ was set to 1,000 (S1 Table). Thus, one might draw different conclusions about the influence of mature forest on this species depending only on the specification of the prior. We note here that the low detection probability for gray jays and Steller’s jays could be a result of a lack of closure (i.e., that they were not always available for detection during a survey); however occupancy models are routinely fit to datasets with similar violations of assumptions (see [28] for a discussion), and with even lower detection probabilities, and thus we believe this example is still illustrative of the issues that can arise from using a prior that is assumed to be non-informative. Models fit with the Logistic or t priors more closely approximated the MLEs than many of the Normal priors, but interestingly did not mirror the MLEs as closely as expected from simulations. Despite the fact that these models did not match the MLEs, the use of these prior did not require iterative model fitting, and thus they should be more generally applicable in occupancy models.

The use of priors that could lead to inferential issues such as those outlined above was common in the recent literature. We found 108 articles published since 2010 that contained the keyphrase “Bayesian occupancy model.” Of the 55 articles reviewed, only 16 articles actually used the model described above (others either did not use occupancy models at all, or fit more complex models, such as multispecies occupancy models). Of these 16, 9 reported using priors on *α*, above, that were incidentally informative on the probability scale. How informative these priors were varied, with some researchers using only moderately informative priors (e.g., Uniform distribution between -8 and 8), and others using priors that were highly informative when transformed to the probability scale (e.g., Normal with a variance of >1,000,000). Further, 4 of the articles did not report their priors or described them only as uninformative. Only 3 articles used priors that were likely to be uninformative, based on our results above. We note, that some researchers did report conducting sensitivity analyses to their priors.

## Discussion and suggested guidance

The results that we present above, combined with the potential prevalence of this issue in the literature, raise concerns about the inference made in regards to species-habitat relationship and resulting distribution patterns. Our literature review, though relatively basic, indicates that this issue might be widespread. Further, most species in a given area are rare [29], meaning that researchers likely are fitting models for species with little data (though this depends on the size of the sampling site relative to the species distribution), which will allow for priors to be more influential. However, the true magnitude of the issue is unknown because the circumstances that allow a seemingly uninformative prior distribution to be in fact informative, can vary, depending on the data. As illustrated in our empirical example, there are scenarios under which the specification of the prior will have negligible impact on inference; however, there also will be times when specifying a prior that does not impact inference will be difficult and require iterative model fitting, whereby models are fit, and posteriors are plotted to assess potential influence of the prior and this process is repeated until inference appears to be unimpacted by the prior. The potential implications of this issue for conservation and management-based studies are significant. We note that camera trapping and the use of occupancy models has become common for studying rare or cryptic, threatened and endangered species [30]. In these studies, both sample sizes and detection probabilities tend to be low, two aspects that can lead to potential issues if informative priors are used. Even small overestimates in occupancy for such species can have major implications for conservation and management action.

It is important to point out that the highlighted issue is not an inherent shortcoming of Bayesian inference. As a rule, Bayesian analysis requires the specification of priors, and as such, inference will be influenced to some degree by these priors. The models fit above are behaving appropriately, and in the above we compared the posteriors to the MLE to illustrate how the priors are influencing results. We do not suggest the MLE is a preferred approach over Bayesian posterior inference, simply that any deviation between the two will be due to the influence of the prior. Priors with large standard deviations or small precisions (inverse of the variance) can strongly influence the posterior distribution of occupancy, both in terms of the most probable value and the shape of the distribution. This is not a unique issue with the basic occupancy model or just the occupancy parameter, but applies more generally to using a Normal prior distribution with a large standard deviation and a non-linear transformation (e.g., Logit, Probit). Importantly, such priors could be commonly used in Bayesian analyses of many types of ecological data, including mark-recapture or survival data.

The issues outlined above bring up a larger philosophical issue of what type of inference researchers want and why they choose to use Bayesian inference. In many cases, integrating informative prior information with new data to update the belief about an ecological process is not only justifiable but philosophically appealing [9]. Informative priors can be particularly useful for the analysis of repeated studies when there is a desire to include information from published research in current analyses, or to borrow strength across data sources to improve estimate precision [31]. Additionally, informative priors can guard against spurious effects [32,33] and erroneous estimation of large effects in underpowered studies [34], thus providing more conservative inference than frequentist analyses. But also, more generally, informative priors and their shrinkage properties provide a coherent form of model selection (i.e., statistical regularization [35]), which has predictive benefits [36]. However, based on our reading of the literature, many ecologists want inference that is free from effects of the prior (e.g., [13,37]). If researchers truly want inference entirely free from any potential influence of the prior, then they likely should look to different frameworks than Bayesian inference (e.g., using likelihood based occupancy models, or for more complex hierarchical models, methods such as data cloning [38]).

For researchers interested in fitting Bayesian occupancy or logistic regression models, we suggest several options. First, following our findings and the recommendation from Hobbs and Hooten [9], researchers that want to use Normal priors should use a variance near 2 (standard deviation approximately 1.4). This prior will often likely be weakly informative when covariates are standardized by subtracting the mean and dividing by the standard deviation. Further, we suggest always conducting a prior sensitivity analysis, where sequentially smaller values of σ are used, and posterior medians and credible intervals are compared so that the extent to which ecological inference is sensitive to prior specification is understood. Second, the Logistic distribution is a good option to ensure priors are weakly informative, again when there are no covariates or when covariates are standardized. It is important to recognize that this prior is not uninformative, just that assuming a Uniform prior for *ψ* or *p* implies an informative prior for its logit [20]. As demonstrated, the Logistic prior can be a robust prior in many situations and often improves MCMC mixing and convergence; an example JAGS model is provided in the Supporting Information (S3 File). Third, set the prior based on the model’s predictive capabilities and hence conduct continuous model selection. Gerber et al. [36] discusses the connection between the Normal prior and ridge regression and the connection between the Laplace prior and LASSO (least absolute selection and shrinkage operator), which are common ways to conduct Bayesian model selection [39]. Fourth, follow the Bayesian paradigm of sequential inferential updating by using previous knowledge to define the prior distribution. In many (sub) fields of ecology there is considerable knowledge available that can be used to define prior distributions. Lastly, we recommend researchers experiment with prior specifications over different scenarios (i.e., *ψ*, *p*, *n*, number of sites, and *σ*) using an easily accessible online tool that we provide for doing so (https://briangerber.shinyapps.io/OccupancyPrior/). This tool allows researchers to investigate prior specifications with simulated data and to upload and analyze their own empirical data.

We further note that recent publications in statistical ecology describing how to fit occupancy models in a Bayesian framework provide some further suggestions for priors that should reduce the concerns we raise here. Kéry and Royle [4], in worked examples suggest, for the intercept, a uniform distribution between 0 and 1 that is then logit-transformed, though with unscaled covariates this prior could lead to estimates of the intercept that are biased low. Further, the Uniform distribution actually leads to an improper posterior, though the degree to which this impacts inference is likely limited.

Beyond the above suggestions, practitioners should use particular care when detection is low, few sites are surveyed, and occupancy is very low or very high. Generally, caution should be applied when parameter uncertainty is likely to be large and near the probability boundaries, 0 and 1. In all cases, but particularly under these circumstances, we suggest first standardizing all independent variables (subtracting the mean and dividing by the standard deviation) so that large magnitude coefficient estimates are avoided ([16]; scaling also speeds convergence in many cases). We caution that surveying many sites is not a panacea for this issue. In the dataset analyzed above, there were 4 visits to over 1,000 sites, a dataset that dwarfs most used in occupancy analyses.

### Conclusion

Complex computational and statistical methods will continue to become more attainable for ecologists and other practitioners as computers become more advanced, and books are published that provide walk-through examples and code to fit complicated models. While the issue of uninformative priors becoming informative when transformed is well known to statisticians [14,15], many of the previous descriptions of this problem are unapproachable for ecologists. We hope that this comment will spur other ecologists to take care to better understand the models that they fit, and what their model outputs and results and the associated ecological inference truly means. The tools are available for us to fit difficult and complex models, the onus is thus on us to understand what they mean.

## Supporting information

S1 Fig Depiction of transformation between logit and probability scale. For each value on the x-axis, which are untransformed, the y-axis is the corresponding value that has been transformed to the probability scale.
(PDF)

S2 Fig Animation of relationship between normal prior distributions on logit and probability scales. Probability density on probability (top panel) and untransformed (bottom panel) scale for normal priors with different values of *σ*^2^ (black boxes).
(PDF)

S3 Fig Supplemental results for gray jay analysis. Posterior distributions (solid lines) of *α* from Bayesian occupancy models with different values for *σ*^2^ on the prior for *α* fit to gray jay data. Maximum likelihood estimate is shown by the dashed vertical line. Panel A presents the posteriors transformed to the probability scale, which equals the estimate of occupancy (*ψ*) when all covariates are held to o (the mean in this example because covariates were centered). Panels B–E presents the posteriors on the untransformed scale.
(PDF)

S1 Table Supplemental results for analysis of avian occupancy. Value of variance (*σ*^2^) for mean 0 Normal prios on *α* and *β* or prior used, and posterior medians for *α*, *β* for the mature forest covariate, *β* for the distance (dist.) to edge covariate, and p for occupancy models fit to data from gray jays (GRJA), Steller’s jays (STJA) and song sparrows (SOSP) in the Oregon Coast Range.
(PDF)

S1 File Example JAGS code to fit occupancy model with covariates. (PDF)

S2 File Example JAGS code to fit occupancy model with no covariates. (PDF)

S3 File Example JAGS code to fit occupancy model with covariates with a logistic distribution. (PDF)
