# Correction: A comment on priors for Bayesian occupancy models

**DOI:** 10.1371/journal.pone.0212346

**Published:** 2019-02-08

**Authors:** Joseph M. Northrup, Brian D. Gerber

Our original paper [1] omitts a reference to a non-peer-reviewed preprint [2] that discusses similar issues. The work by Lele [2] illustrates how priors in Bayesian analysis can impact inference in general and provides cautionary advice about the use of non-informative priors in analyses used for wildlife management. Similar to our paper, Lele [2] shows how seemingly non-informative priors can influence inference in occupancy models. Our work [1] builds on this research by further exploring this issue with simulation and an empirical example, describing in plain language the reasons that seemingly non-informative priors can influence inference, and providing potential alternative priors that can be used to provide the inference often desired by researchers.
