# Supplementary material for: A comment on priors for Bayesian occupancy models
Source: PLoS One. 2018 Feb 26;13(2):e0192819. doi: 10.1371/journal.pone.0192819 (PMC5826699; doi:10.1371/journal.pone.0192819)
Supplement: S1 Fig — For each value on the x-axis, which are untransformed, the y-axis is the corresponding value that has been transformed to the probability scale. (PDF) [file pone.0192819.s001.pdf]

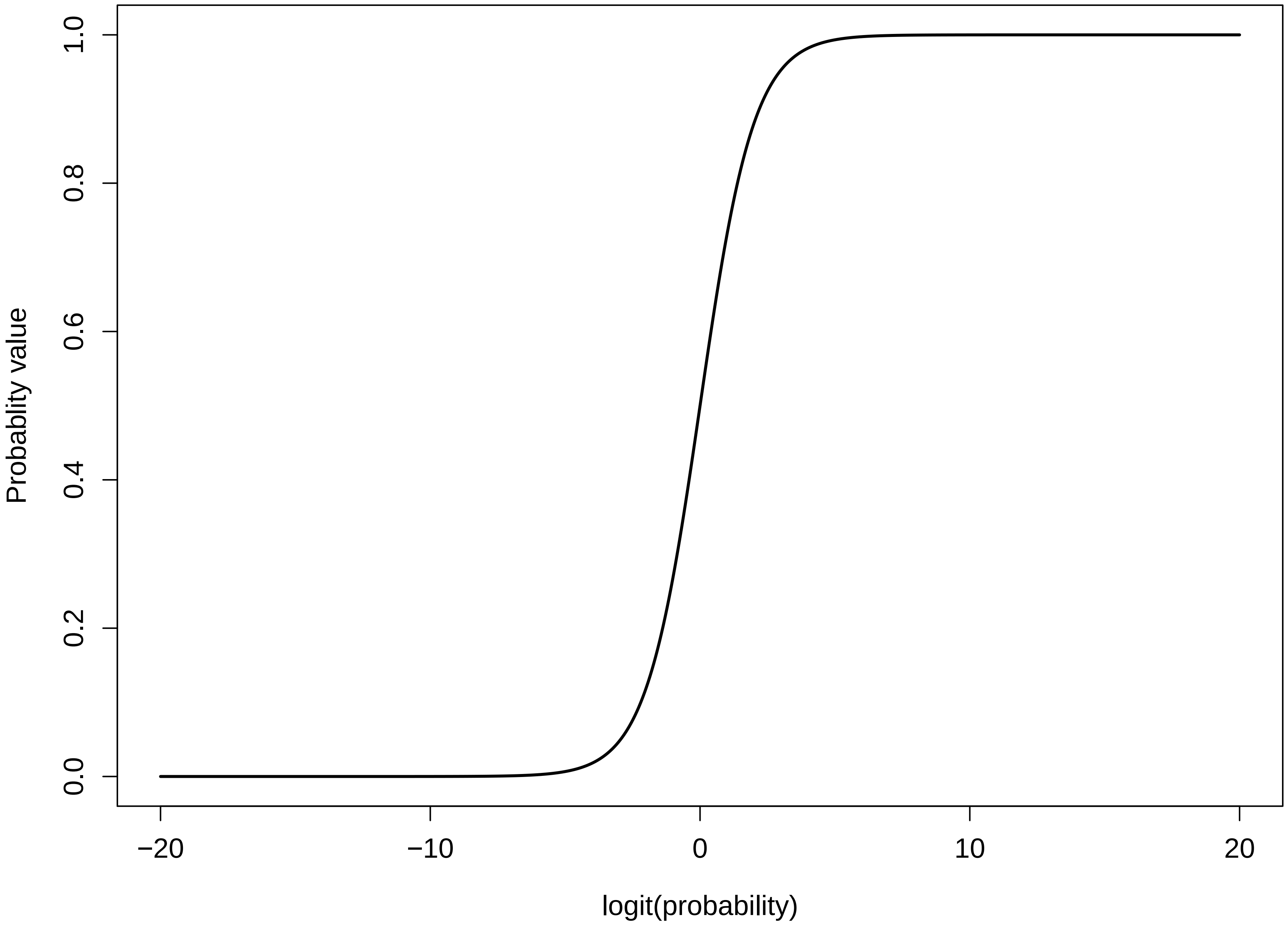

Probablity value
logit(probability)
0.0
0.2
0.4
0.6
0.8
1.0
−20
−10
0
10
20

Figure. S1. For each value on the x-axis, which are untransformed the y-axis is the corresponding value that has been transformed to the probability scale.
