# Supplementary material for: A comment on priors for Bayesian occupancy models
Source: PLoS One. 2018 Feb 26;13(2):e0192819. doi: 10.1371/journal.pone.0192819 (PMC5826699; doi:10.1371/journal.pone.0192819)
Supplement: S3 Fig — Posterior distributions (solid lines) of α from Bayesian occupancy models with different values for σ2 on the prior for α fit to gray jay data. Maximum likelihood estimate is shown by the dashed vertical line. Panel A presents the posteriors transformed to the probability scale, which equals the estimate of occupancy (ψ) when all covariates are held to o (the mean in this example because covariates were centered). Panels B–E presents the posteriors on the untransformed scale. (PDF) [file pone.0192819.s003.pdf]

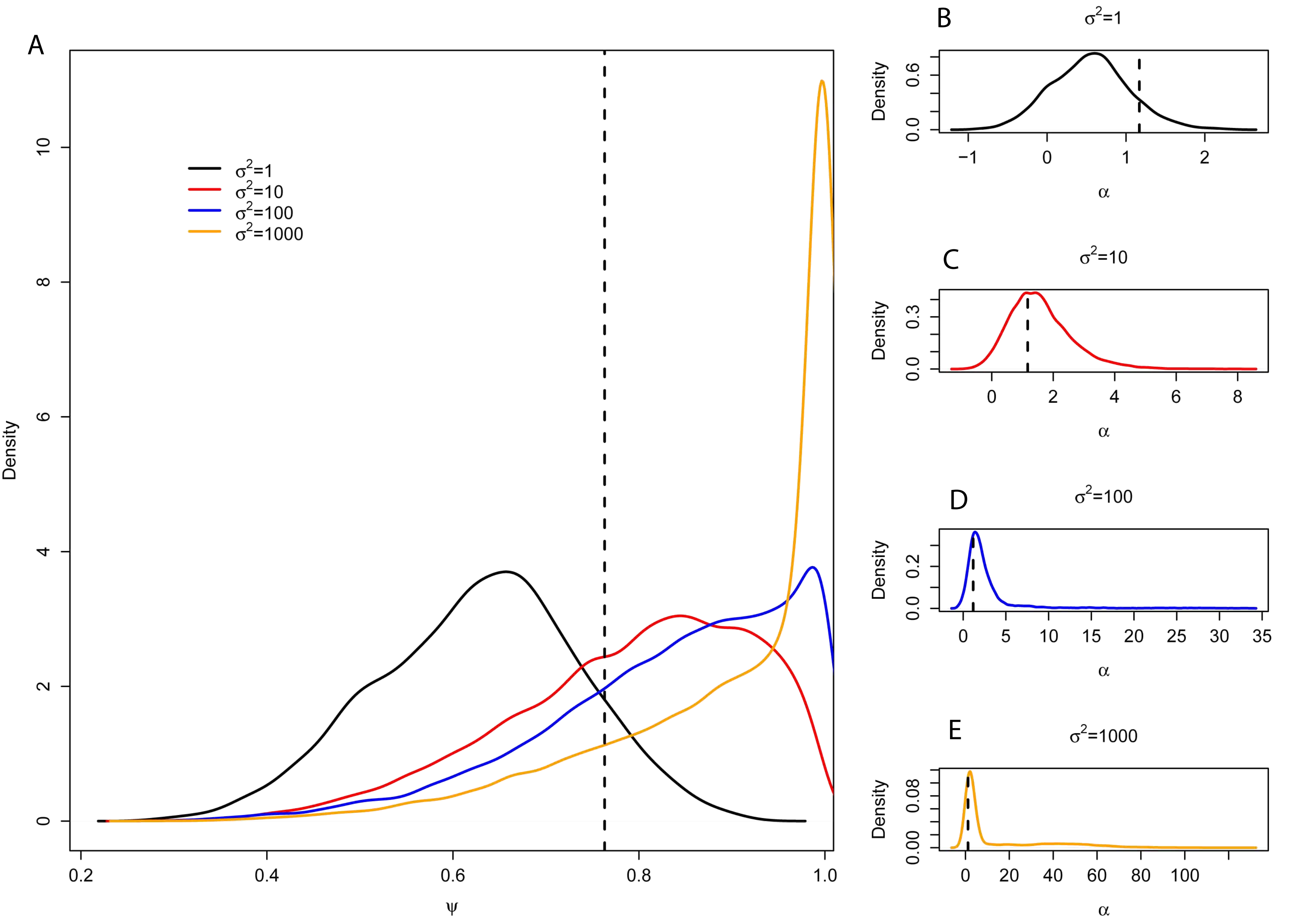

A
B
C
D
E
Density
ψ
α
$\sigma^2=1$
$\sigma^2=10$
$\sigma^2=100$
$\sigma^2=1000$
0.2
0.4
0.6
0.8
1.0
0
2
4
6
8
10
0.0
0.6
−1
1
0.3
0.2
5
15
20
25
30
35
0.00
0.08
40
60
80
100

Figure S3. Posterior distributions (solid lines) of α from Bayesian occupancy models with different values for σ^2 on the prior for α fit to gray jay data. Maximum likelihood estimate is shown by the dashed vertical line. Panel A presents the posteriors transformed to the probability scale, which equals the estimate of occupancy (ψ) when all covariates are held to 0 (the mean in this example because covariates were centered). Panels B – E presents the posteriors on the untransformed scale.
