# Supplementary material for: A comment on priors for Bayesian occupancy models
Source: PLoS One. 2018 Feb 26;13(2):e0192819. doi: 10.1371/journal.pone.0192819 (PMC5826699; doi:10.1371/journal.pone.0192819)
Supplement: S1 Table — Value of variance (σ2) for mean 0 Normal prios on α and β or prior used, and posterior medians for α, β for the mature forest covariate, β for the distance (dist.) to edge covariate, and p for occupancy models fit to data from gray jays (GRJA), Steller’s jays (STJA) and song sparrows (SOSP) in the Oregon Coast Range. (PDF) [file pone.0192819.s004.pdf]

Table S1. Value of variance (σ^2) for mean 0 Normal priors on α and β or prior used, and posterior medians for α, β for the mature forest covariate, β for the distance (dist.) to edge covariate, and p for occupancy models fit to data from gray jays (GRJA), Steller's jays (STJA) and song sparrows (SOSP) in the Oregon Coast Range.

Species	σ^2 for prior on α and β	Posterior median α (lower and upper credible interval)	Posterior median β for mature forest (lower and upper credible interval)	Posterior median β for dist. edge (lower and upper credible interval)	Posterior median p (lower and upper credible interval)
GRJA					
	1000	3.09 (0.25, 67.85)	2.46 (-9.24, 27.55)	0.38 (-5.23, 30.51)	0.03 (0.02, 0.05)
	100	1.82 (0.04, 14.71)	2.10 (0.43, 5.73)	0.31 (-0.36, 6.58)	0.04 (0.03, 0.06)
	10	1.42 (-0.05, 3.69)	1.84 (0.68, 3.62)	0.258 (-0.28, 1.48)	0.04 (0.03, 0.06)
	1	0.6 (-0.34, 1.83)	1.20 (0.51, 2.2)	0.19 (-0.23, 0.78)	0.05 (0.03, 0.07)
	MLE	1.17 (-0.56, 2.90)	1.76 (0.22, 3.32)	0.23 (-0.30, 0.77)	0.04 (0.03, 0.06)
	Logistic	1.04 (-0.15, 2.54)	1.54 (0.6, 2.87)	0.25 (-0.23, 0.93)	0.04 (0.03, 0.06)
	t	0.91 (-0.25, 2.39)	1.46 (0.55, 2.78)	0.23 (-0.26, 0.96)	0.04 (0.03, 0.07)
STJA					
	1000	43.99 (12.09, 89.80)	1.76 (-16.93, 33.18)	5.1 (-13.44, 36.83)	0.08 (0.07, 0.09)
	100	14.57 (4.82, 29.22)	0.21 (-5.82, 10.78)	1.44 (-5.15, 12.87)	0.08 (0.07, 0.09)
	10	4.7 (2.06, 9.34)	-0.11 (-2.31, 2.89)	-0.36 (-1.99, 3.56)	0.08 (0.07, 0.1)
	1	1.80 (0.92, 2.99)	0.01 (-0.65, 0.83)	-0.30 (-0.87, 0.46)	0.1 (0.08, 0.12)
	MLE	2.99 (-3.33, 9.32)	-0.49 (-2.99, 2.02)	-0.87 (-2.99, 1.25)	0.09 (0.07, 0.12)
	Logistic	2.73 (1.16, 6.17)	-0.03 (-1.24, 1.58)	-0.37 (-1.3, 1.46)	0.09 (0.08, 0.11)
	t	2.86 (1.26, 7.31)	-0.03 (-1.24, 1.78)	-0.38 (-1.36, 1.78)	0.09 (0.08, 0.11)
SOSP					
	1000	-1.02 (-1.17, -0.86)	-0.53 (-0.70, -0.36)	-0.36 (-0.52, -0.2)	0.47 (0.44, 0.51)
	100	-1.02 (-1.17, -0.87)	-0.53 (-0.70, -0.36)	-0.36 (-0.52, -0.2)	0.47 (0.44, 0.51)
	10	-1.02 (-1.17, -0.87)	-0.53 (-0.70, -0.36)	-0.36 (-0.52, -0.2)	0.47 (0.44, 0.51)
	1	-1.01 (-1.17, -0.86)	-0.52 (-0.70, -0.36)	-0.35 (-0.52, -0.2)	0.47 (0.44, 0.51)
	MLE	-1.01 (-1.17, -0.86)	-0.52 (-0.69, -0.36)	-0.35 (-0.51, -0.19)	0.47 (0.44, 0.51)

Logistic	-1.01 (-1.18, -0.85)	-0.53 (-0.70, -0.36)	-0.35 (-0.51, -0.19)	0.47 (0.44, 0.51)
t	-1.02 (-1.18, -0.86)	-0.52 (-0.69, -0.35)	-0.35 (-0.52, -0.19)	0.47 (0.44, 0.51)
