# Supplementary material for: A comment on priors for Bayesian occupancy models
Source: PLoS One. 2018 Feb 26;13(2):e0192819. doi: 10.1371/journal.pone.0192819 (PMC5826699; doi:10.1371/journal.pone.0192819)
Supplement: S1 File — (PDF) [file pone.0192819.s005.pdf]

Below is an example of JAGS code used to fit a simple occupancy model with covariates

```
model {
	# Priors
	b0~dnorm(0,1)		#Intercept
	b1~dnorm(0,1)		#Beta_1
	b2~dnorm(0,1)		#Beta_2
	p~dunif(0,1)		#Detetcion


	for(i in 1:n.sites){
		psi[i]<-exp(b0+b1*x1[i]+b2*x2[i])/(1+exp(b0+b1*x1[i]+b2*x2[i]))

		z[i]~dbin(psi[i],1)

		mu[i]<-p*z[i]

		y[i]~dbin(mu[i],n.occs)
	}
}
```
