# Supplementary material for: A comment on priors for Bayesian occupancy models
Source: PLoS One. 2018 Feb 26;13(2):e0192819. doi: 10.1371/journal.pone.0192819 (PMC5826699; doi:10.1371/journal.pone.0192819)
Supplement: S3 File — (PDF) [file pone.0192819.s007.pdf]

Below is an example of JAGS code used to fit a simple occupancy model with covariates and a logistic distribution prior

```
model {
        # Priors
        b0~dlogis(0,1)
        p~dunif(0,1)

        #model
        for(i in 1:n.sites){
         psi[i]<-exp(b0)/(1+exp(b0))
                z[i]~dbin(psi[i],1)
                mu[i]<-p*z[i]
                y[i]~dbin(mu[i],n.occs)
        }
}
```
