# Supplementary material for: A comment on priors for Bayesian occupancy models
Source: PLoS One. 2018 Feb 26;13(2):e0192819. doi: 10.1371/journal.pone.0192819 (PMC5826699; doi:10.1371/journal.pone.0192819)
Supplement: S2 Fig — Probability density on probability (top panel) and untransformed (bottom panel) scale for normal priors with different values of σ2 (black boxes). (PDF) [file pone.0192819.s002.pdf]

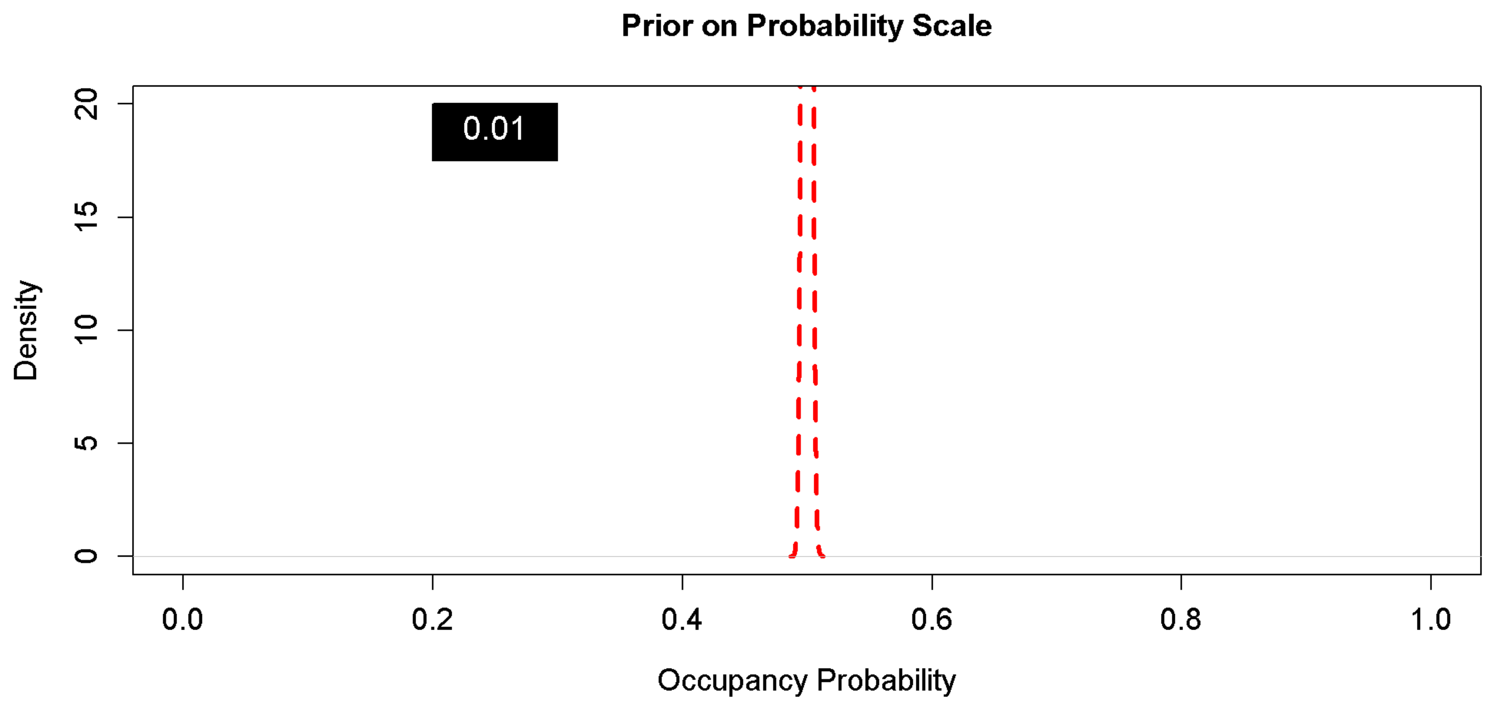
Prior on Probability Scale
0.01
Density
0
5
10
15
20
0.0
0.2
0.4
0.6
0.8
1.0
Occupancy Probability

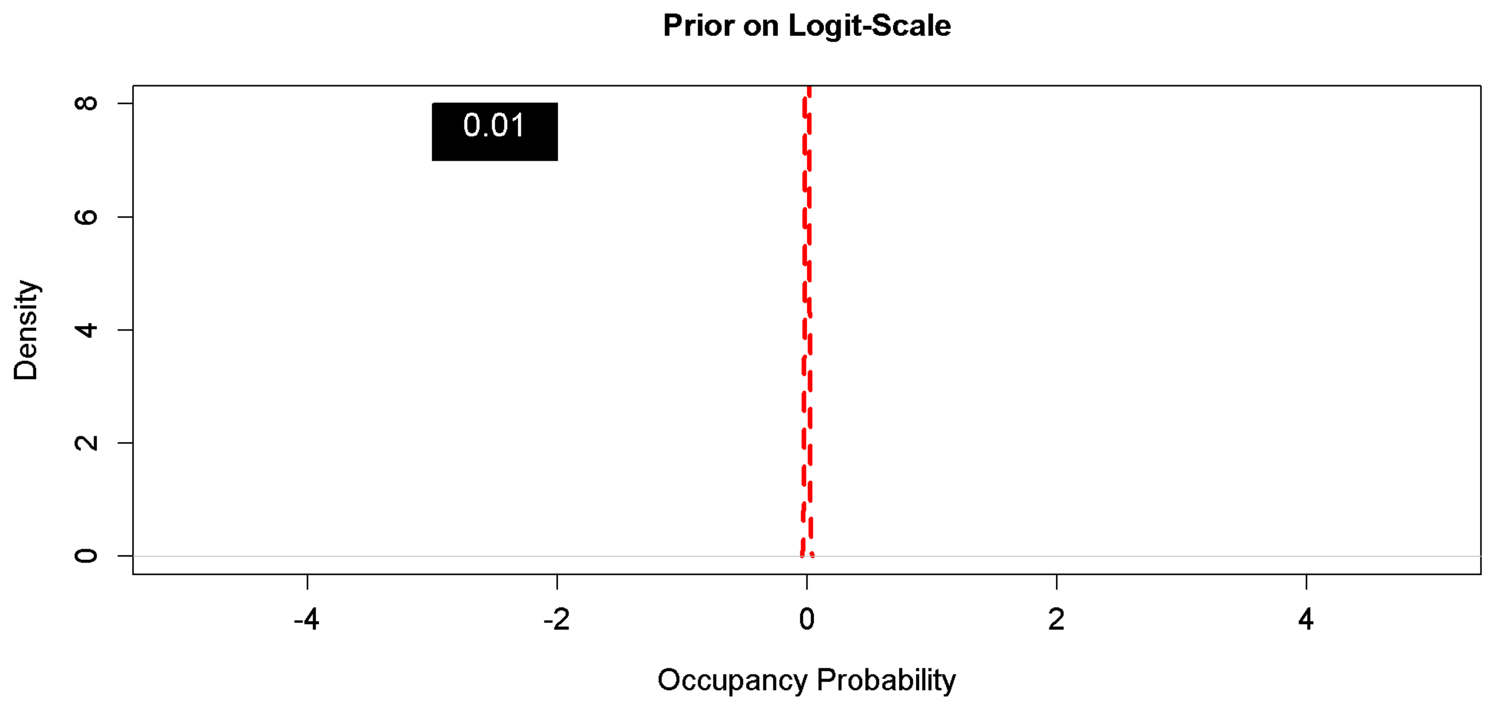
Prior on Logit-Scale
0.01
Density
0
2
4
6
8
-4
-2
0
2
4
Occupancy Probability
